# Periodontal bacterial supernatants modify differentiation, migration and inflammatory cytokine expression in human periodontal ligament stem cells

**DOI:** 10.1371/journal.pone.0219181

**Published:** 2019-07-03

**Authors:** Liza L. Ramenzoni, Giancarlo Russo, Maria D. Moccia, Thomas Attin, Patrick R. Schmidlin

**Affiliations:** 1 Clinic of Conservative and Preventive Dentistry, Center of Dental Medicine, University of Zurich, Zurich, Switzerland; 2 Laboratory of Applied Periodontal and Peri-implantitis Sciences, Clinic of Conservative and Preventive Dentistry, Center of Dental Medicine, University of Zurich, Zurich, Switzerland; 3 Functional Genomics Center Zurich, ETH, University of Zurich, Zurich, Switzerland; INSERM, FRANCE

## Abstract

Periodontal ligament stem cells (PDLSC) play an important role in periodontal tissue homeostasis/turnover and could be applied in cell-based periodontal regenerative therapy. Bacterial supernatants secreted from diverse periodontal bacteria induce the production of cytokines that contribute to local periodontal tissue destruction. However, little is known about the impact of whole bacterial toxins on the biological behavior of PDLSC. Therefore this study investigated whether proliferation, migration, inflammatory cytokines expression and transcriptional profile would be affected by exposure to endotoxins from bacterial species found in the subgingival plaque. PDLSC were cultured with the following bacterial supernatants: *S*. *mutans*, *S*. *anginosus*, *P*. *intermedia*, *F*. *nucleatum*, *P*. *gingivalis* and *T*. *denticola*. These supernatants were prepared in dilutions of 1:1000, 1:500, 1:300 and 1:50. Using quantitative RT-PCR, gene expression of selected inflammatory cytokines (*IL-6*, *IL-8* and *IL-1β*) and cell-surface receptors (*TLR2*, *TLR4*) showed upregulation of ≈2.0- to 3.0-fold, when exposed to *P*. *intermedia*, *F*. *nucleatum*, *P*. *gingivalis* and *T*. *denticola*. However, supernatants did not affect proliferation (MTT) and migration (wound scratch assays) of PDLSC. Next generation RNA sequencing confirmed modified lineage commitment of PDLSC by stimulating chondrogenesis, adipogenesis and inhibition of osteogenesis under *P*. *gingivalis* supernatant treatment compared to control. Taken together, this study shows stem cell immunomodulatory response to different periodontal bacteria supernatant and suggests that stem cell transcriptional capacity, migration/proliferation and osteogenesis may differ in the presence of those pathogens. These results bring into question stem cell contribution to periodontal tissue regeneration and onset of inflammation.

## Introduction

Periodontitis is a chronic inflammatory disease that leads to the destruction of periodontal tissues and subsequent tooth loss, mainly due to subgingival bacterial infection [1, 2]. Endotoxins secreted from pathogenic bacteria are crucial virulence factors involved in the initiation and establishment of periodontitis, stimulating proinflammatory cytokine production, monocyte/lymphocyte infiltration and bone resorption [3, 4]. In both healthy and pathological conditions, periodontal ligament stem cells (PDLSCs) play an important role in maintaining homeostasis and inducing tissue regeneration owing to their ability to differentiate into major cellular types, such as osteoblasts, fibroblasts and cementoblasts [5–7]. More specifically, studies have demonstrated that due to their osteogenic potential, PDLSCs have the capacity not only to regenerate and repair alveolar bone tissues [8], but also to modulate differentiation and immunomodulatory characteristics of the periodontal tissue [9]. Endotoxins produced by periodontal pathogens may interact with progenitor periodontal stem cells, which could either result in tissue homeostasis and resolution of inflammation, or further progression of periodontitis.

To better understand the impact of inflammatory bacterial endotoxins on PDLSCs differentiation, more knowledge is required. Some reports have revealed stimulating [10] or inhibiting effects of purified lipopolysaccharides (LPS) on the differentiation potential of PDLSCs and changing cell fate by enhancing the production of IL-6 and IL-8 [11]. Moreover, it has been suggested that *P*. *gingivalis* LPS could induce the expression of pro-inflammatory cytokines in periodontal ligament cells, and thus lead to disturbance of the differentiation of dental stem cells [11–14]. Even though PDLSCs may play a role in the immune inflammatory response [10, 15], the interaction of different periodontopathogenic endotoxins with PDLSCs has never been fully investigated. Toll-like receptors -2 (TLR2) and -4 (TLR4) have been identified as signaling receptors of bacterial endotoxins [12] but the PDLSCs capacity to express TLRs for endotoxin still need further investigation. Additionally, potential migration of stem cells and their possible innate immune response against bacterial endotoxins could play an important role in their possible therapeutic use on periodontal treatments [16–18]. Therefore, the success of regenerative periodontal treatment could be improved by understanding the migration and cytokine secretion of PDLSCs when exposed to periodontal bacterial endotoxins.

Accordingly, the present study was undertaken to provide new insight into the impact of periodontal bacterial endotoxins from different periodontal bacteria on the pro-inflammatory cytokine production and functional properties of PDLSCs. PLDSCs immunoresponse and differentiation capacity in the presence of culture supernatants collected from different periodontal bacteria species was analyzed. Additionally, cell proliferation and migratory efficacy of endotoxin-treated PDLSC was examined.

## Materials and methods

### Isolation, cultivation and phenotype analysis

Disease free impacted third molars were obtained from 3 adult donors patients undergoing tooth extraction for orthodontic or therapeutic reasons at Department of Oral Surgery of the Center of Dental Medicine, University of Zurich. Approval to conduct this study was granted by the Swiss ethics committees for research involving humans (2016–00243) and informed consent obtained from the donors, in accordance with the Declaration of Helsinki. PDLSCs were isolated and cultured following previous study methodology [11]. Periodontal ligament cells were scraped from these third molars and then enzymatically digested for 30 min at 37°C in a solution of collagenase/dispase (Sigma, St. Louis, MO, USA). Cells were cultured in Dulbecco's modified Eagle's medium (DMEM), supplemented with 10% fetal bovine serum (FBS), streptomycin (50 μg/ml) and penicillin (100 U/ml) at 37°C in a 5% CO_2_ humidified atmosphere. The cells were then seeded onto T75 culture dishes (Falcon BD) and incubated at 37°C in 5% CO_2_. Cells at passage P3–P6 were used in the experiments. For the phenotype analysis, primary PDLSC were stained with monoclonal antibodies STRO-1, CD105, CD166 and CD90-biotin IgM antibody (eBiosciences, San Diego, CA, USA; 2 lg/ml) for 1 h on ice. A goat anti-human IgM FITC secondary antibody (Molecular Probes, Eugene, Carlsbad, CA, USA; 2 lg⁄ml) was incubated for 1 h. Cell surface-marker STRO-1, CD105, CD166 and CD90 positive cells (Table 1) were analyzed and sorted by fluorescence-activated cell sorting in a flow cytometry facility (FACSCalibur, Becton Dickinson, CA, USA).

**Table 1:** Flow cytometric analysis of cell surface expression of STRO-1, CD105, CD166 and CD90. One million PDL cells were analyzed with antibodies against the human antigens STRO-1, CD105, CD166 and CD90 by flow cytometry. Positive expression was defined as the level of fluorescence greater than 99% of the corresponding isotype-matched control antibodies. Data represent (%) mean ± standard deviation (SD) (n = 3).

Marker	Percentage positivity
**STRO-1**	92.1 ± 33.5%
**CD105**	96.5 ± 10.8%
**CD166**	67.2 ± 19.1%
**CD90**	84.5 ± 35%

### Bacterial strains and supernatants

Six bacterial species were used in this study: *Streptococcus mutans* UAB159 (OMZ 918), *Streptococcus anginosus* ATCC9895T (OMZ 871), *Prevotella intermedia* ATCC25611T (OMZ 278), *Fusobacterium nucleatum* KP-F8 (OMZ 598), *Porphyromonas gingivalis* ATCC33277T (OMZ 308) and *Treponema denticola* ATCC35405T (OMZ 661). *Treponema denticola* was cultivated under anaerobic conditions in 10 mL of spirochetes medium OMIZ-W68 and cultivated anaerobically at 37°C. The other strains were cultured on Columbia Blood Agar (CBA) plates under the same anaerobic conditions. Liquid pre-cultures were prepared by inoculation of the bacterial colonies from the Columbia Blood Agar (CBA) plates into modified fluid universal medium (mFUM). The growth was obtained for 3 to 4 days at 37°C, followed by further anaerobic sub-culturing for 2 to 3 days at 37°C in Brain Heart Infusion broth, containing 0.5% hemin and 0.2% menadione. After incubation, the total of bacterial counts per bacterial species were determined using a stereomicroscope at 16× magnification (Zeiss, Oberkochen, Germany). The bacterial cultures were grown to the same density (approximately 5 × 10^7^ CFU/ml, supporting information S1 Fig) and were adjusted to OD550 nm = 1.0. Correspondence was established between OD 550 nm and bacteria counted in microscopy. Then, they were centrifuged at 10.000 × g for 10 min at 4°C. The supernatants were finally filter–sterilized (0.2μm filter) to free them from bacterial debri/cells and stored at −80°C. The concentration of protein content of all supernatants was measured with Qubit Protein Assay Kit (Life Technologies). Finally, for stimulation experiments, the bacterial supernatants were directly diluted in the cell culture medium of the PDLSCs to provide distinct working solutions with final dilution of 1:1000, 1:500, 1:300 and 1:50 [19] for comparison among each bacterial species for 24 h. Cells treated with phosphate-buffered saline (PBS 1X) were used as negative control (untreated cells). Based on the collection method used here and other studies [19–21], the expected virulence factors present in the bacterial supernatants in the present study are as follows: LPS, fimbriae, lipd-A associated proteins, hemagglutinin, hemolysins, peptidoglycan, DNA, cystein proteases (gingipains), trypsin-like protease, outer membrane vesicles, sialidade and metalloproteases. However, supernatant components were not specifically identified or quantified for the series of experiments in this study.

### Cell viability and proliferation

PDLSCs viability was determined by thiazolyl blue tetrazolium (MTT; Sigma–Aldrich) dye reduction assay (5 mg/ml in phosphate buffered saline). 5 × 10^3^ cells/well were seeded in 96-well plates. At 24, 48 and 72 h after exposure to the 1:1000, 1:500, 1:300 and 1:50 dilutions of *P*. *gingivalis* and *T*. *denticola*, 500 ml of MTT was added to each well and incubated for 4 h at 37°C in the dark. In the next step, MTT was removed by aspiration from the wells and isopropanol was added (200 ml; 1 N HCl) to solubilize the MTT formazan crystals formed. The untreated control was used as the positive control and as reference for the sample cell viability. Absorbance was measured in optical density at a wavelength of 570 nm with an ELISA plate spectrophotometer reader (Quant Bio-Tek Instruments Inc., Winooski, USA). The following formula was used to calculate cell viability: Cell viability (%) = (OD of sample / OD of positive control) x 100. For estimation of PDLSCs proliferation, cells were seeded at concentration 2 × 10^4^ cells/well in 24-well plates and cultivated in standard conditions. After 24 h seeding, cells were treated with 1:1000, 1:500, 1:300 and 1:50 of supernatant dilutions for further 24, 48 and 72 h. For cell proliferation assays, cell numbers were counted using flow cytometry at different times post-treatment. Cells were centrifuged and re-suspended in 100 μl of ice-cold PBS containing flow cytometry buffer. Afterwards, 20 μl of fluorescein isothiocyanate (FITC)-conjugated monoclonal anti-human STRO-1 (eBiosciences, San Diego, CA, USA) were added to this cell suspension and incubated on ice in the dark for 30 min. The cells were washed twice after staining, finally re-suspended in 0.2 ml of flow cytometry buffer and measured by flow cytometry (FACSCalibur, Becton Dickinson, CA, USA). Each sample was acquired in triplicate. For data analyses the software CellQuest 3.3 (Becton Dickinson, Franklin Lakes, NJ, USA) was used.

### Cell migration assay

Cell migration was evaluated by a scratch wound assay where PDLSC were seeded at concentration of 2 × 10^4^ cells/well in 24-well plates and cultivated under serum starvation to maximal 60% of confluence. The scratch was produced 16 hours after the beginning of serum starvation of the cells, to halt proliferative response during wound closure. Next, each well was wounded by scratching with a 10 μl pipette tip. In each well, 3 different scratches were performed and images from all scratches were considered on the results. Following PBS washes to remove cell debris, the cultures were exposed to *P*. *gingivalis* and *T*. *denticola* supernatant dilutions (1:1000, 1:500, 1:300 and 1:50). Cell counting was also performed before and after the closure to assure low change in cell number between the start and end of the experiment. Digital images were captured using a camera-equipped, inverted microscope (Carl Zeiss, Inc., Thorwood, NY, USA) using the LAS-X Leica image acquisition software (Leica Microsystems, Wetzlar, Germany) and wound width measurements were subtracted from wound width at time zero to obtain the net wound closure. The images of the migration assay were obtained from the exact same position within the assay well. The distance between edges of the injured monolayer was measured by Image J software (Software 1.48q, Rayne Rasband, National Institutes of Health, USA) in pixels and wound closure areas were expressed as the difference in width at 0 h, 12 h and 24 h after wound simulation by subtracting the total amount of greyscale pixel counted in the cell-free area remaining after 24 h from the initial wound area (wound closure area). Since the scratch width varied to some extent from one wound to the other, a “relative wound closure” (RWC) area was calculated by normalizing the measured wound closure area (in pixels) to the total area of the image, which is covered in pixels (RWC [%] = wound closure area [pixel] × 100 [%]/× [pixel]. Assays were performed three times on independent days using triplicate wells.

### Quantitative real time PCR

Gene expression analysis was conducted using PDLSCs that were seeded at concentration of 2 × 10^4^ cells/well in 24-well plates with medium described above, then treated with collected bacterial supernatants in dilutions (control [untreated], 1:1000, 1:500, 1:300 and 1:50) for 24 h. Total RNA was extracted from the cells by Trizol Reagent (Gibco, Life Technologies, Carlsbad, CA, USA). DNase I (AM2238, Invitrogen, Carlsbad, USA) was added to RNA samples and incubated at 37°C for 30 min to degrade DNA in the presence of RNA. Then, RNA was quantified using NanoDrop ND-1000 (Thermo-Fisher Scientific, Wohlen, Switzerland). cDNA was synthesized using an iScript kit (Bio-Rad, Hercules, CA, USA). Quantitative real time PCR (RT-PCR) reactions were carried out on a CFX96 real-time PCR system (Bio-Rad) by initial incubations of 2 min at 50°C and 10 min at 95°C, followed by 40 cycles of 15 s at 95°C and 1 min at 60°C and run in a total reaction volume of 15 μL, containing 7.5 μL of SYBRGreen PCR Master Mix (LifeTechnologies, Zug, Switzerland), 6 μL of sample (1ng) and 1.5 μL of primer solution of 1 μM (mixture of forward and reverse primers). Gene expression levels were determined by RT-PCR for innate/adaptive immunity response for the following genes: *TLR2*, *TLR4*, *IL-6*, *IL-8* and *IL-1β*. Three independent experiments were performed for all genes for human *GAPDH* (forward primer: 5-AATCCCATCACCATCTTCCA-3, reverse primer: 5'-TGGACTCCACGACGTACTCA-3', *IL-6* (foward primer: 5'-GGTACATCCTCGACGGCATCT-3', reverse primer: 5'-GTGCCTCTTTGCTGCTTTCAC-3'), *IL-8* (foward primer: 5'-ATGACTTCCAAGCTGGCCGTGGCT-3', reverse primer: 5'- TCTCAGCCCTCTTCAAAAACTTCTC-3'), *IL-1β* (foward primer: 5'- ACAGATGAAGTGCTCCTTCCA-3', reverse primer: 5'- GTCGGAGATTCGTAGCTGGAT -3'), *TLR2* (foward primer: 5'-GCTCTCTGCTCCTCCCTGTT-3', reverse primer: 5'- CACACCGACCTTCACCATCT-3'), *TLR4* (foward primer: 5'- CAGAGTTGCTTTCAATGGCATC-3', reverse primer: 5'- AGACTGTAATCAAGAACCTGGAGG-3'). From the Cq values obtained with the qPCR, the expression levels of transcripts were calculated by using the comparative Ct method (2^−ΔΔCT^ formula) after normalization to the internal reference gene (*GAPDH*). The results are presented in means ± standard deviations.

### Enzyme-linked immunosorbent assay (ELISA)

PDLSC linked immunosorbent assay (ELISA) read after 24 h in 96-well flat-bottomed plates in medium described above. Cells were grown until confluent monolayers, followed by stimulation with dilutions of 1:500 and 1:50 of *P*. *gingivalis* bacterial supernatant for further 24 h. Untreated cells were considered as control. The supernatants collected were centrifuged for 10 min at 1000g (4°C) to remove cell debris. The concentration of the cytokines was measured using commercially available ELISA kits for IL-1β (ab46052) and IL-6 (ab46027) (Abcam, Cambridge, UK) according to the instructions provided by the manufacturer. Absorbance was measured at a wavelength of 450 nm using a microplate reader (Bio-Rad Laboratories). Values were calculated on the basis of a standard curve constructed for each assay.

### Western blot analysis

PDLSCs were seeded at concentration of 2 × 10^4^ cells/well in 24-well plates and cultured with dilutions of 1:500 and 1:50 of *P*. *gingivalis* supernatant for 24 h. Untreated standard media was used as control. Then, cells were solubilized with radioimmunoprecipitation assay lysis buffer (Thermo Fisher Scientific, Inc.) and protein concentration was measured using a BCA kit (Thermo Fisher Scientific, Inc.). Approximately 50 μg of protein from each sample was separated by 12% SDS-PAGE and transferred to a polyvinylidene fluoride membrane (EMD Millipore, Burlington, MA, USA). Following blocking with 5% bovine serum albumin (1:100; Sigma-Aldrich; Merck KGaA) in Tris-buffered saline with Tween for 2 h at room temperature, membranes were incubated with TLR2 (ab191458) and TLR4 (ab13556) primary antibodies (Abcam, Cambridge, UK) at a dilution of 1:1000 overnight at 4°C followed by incubation with anti-TLR4 antibody (Biotin) (ab183459) and anti-TLR2 antibody (ab191458) (at a dilution of 1:2000; Abcam) for 1 h at room temperature. GAPDH (ab80863); dilution 1:2000; Abcam) was used as an internal control. Protein blots were visualized and analyzed using a chemiluminescence system (Bio-Rad Laboratories, Inc., Hercules, CA, USA) and autoradiography films (Kodak Image Station 440; Kodak, Rochester, NY, USA). Relative concentration of proteins was quantitated using the Image J Software (National Institutes of Health, Bethesda, USA).

### Alizarin red staining

The PDLSCs osteogenic differentiation was assessed using Alizarin red S (Sigma-Aldrich) staining as a biochemical mineralization assay. Cells were seeded in 24-well culture plates at a density of 2 × 10^4^ cells/well to confluence in normal medium. Osteogenic medium was α-MEM supplemented with 10% FBS, 100 nM dexamethasone, 5 mM β-glycerophosphate and 50 μg/ml L-ascorbic acid (Sigma-Aldrich). Experimental group cells were cultured for 21 days in induction medium additionally supplemented with dilutions of 1:500 and 1:50 of *P*. *gingivalis*. The induction medium was changed every three days. Cell fixation was performed using 4% paraformaldehyde for 10 min at 4°C. Before the staining, fixed cells were washed twice with deionized water. Then, 1 ml of 1% w/v Alizarin Red S solution (Sigma-Aldrich) was added to each well for 10–15 min, followed by washing with deionized water before imaging. For Alizarin Red S staining quantification, stained cells were washed with deionized water and incubated with 200 ml of destaining solution (20% methanol, 10% acetic acid in deionized water) during 15–20 min before measuring the absorbance of the solution at 405 nm in the microplate reader.

### RNA sequencing analysis

PDLSCs were seeded at concentration of 2 × 10^4^ cells/well in 24-well plates and cultured in DMEM containing 10% FCS/1% antibiotics with or without dilutions of 1:500 and 1:50 of *P*. *gingivalis* supernatants for 24 h. For RNA sequencing (RNAseq), 3 samples with 1:500 treatments, 3 samples with 1:50 treatment and 3 control samples (without supernatant treatment) underwent total RNA isolation using a RNA Cell Miniprep System (Promega, USA). TruSeq RNA Access library kit (Illumina, Inc., San Diego, CA, USA) was applied to make RNA sequencing libraries and samples were analyzed at the Functional Genomics Center Zurich (FGCZ), ETH Zurich, University of Zurich. The quality and quantity of the enriched libraries were validated using Qubit (1.0) Fluorometer and the Caliper GX LabChip GX (Caliper Life Sciences, Inc., USA). The product was a smear with an average fragment size of approximately 260 bp. Sequencing were performed on the Illumina HiSeq 4000 single end 125 bp using the TruSeq SBS Kit HS4000 (Illumina, Inc, California, USA) according to standard protocols used at the FGCZ. To identify the lineage commitment of *P*. *gingivalis* treated PDLSC, focus was placed on identifying any variations in the expression of genes involved in inflammation (confirmation of RT-PCR results: *TLR2*, *TLR4*, *IL-6*, *IL-8* and *IL-1β*), osteogenesis, chondrogenesis and adipogenesis. Differentially expressed genes were identified using the R package edgeR from Bioconductor Version 3.0. Gene Ontology (GO) clustering was analyzed by using the online Database for Annotation, Visualization and Integration Discovery (DAVID) bioinformatics resources, version 6.7 (http://david.abcc.ncifcrf.gov).

### Statistical analysis

Statistical analysis was completed using IBM SPSS software (IBM SPSS Statistics for Windows, version 23.0; IBM Corp., Armonk, NY). For the RT-PCR analysis, linear mixed model statistics were performed to simultaneously explore the effect of the different bacterial supernatants, the different dilutions and the genes examined, as simultaneous models usually increase the power of the statistical analysis compared to a large series of pairwise comparisons. The group differences were identified using post hoc tests with a Bonferroni correction for multiple comparisons. It was assumed that repeated measurements taken from the same bacterial supernatant with the same dilution are correlated. Quantitative RT-PCR data was presented together with the significance of paired-tests between the different configurations of genes, bacterial supernatants and dilution. The quantitative data is presented as mean ± standard deviation (SD); p ≤ 0.05 was considered to be statistically significant. For cell viability, proliferation and migration, analysis of variance (ANOVA) was used to show significant differences in results followed by the post hoc Fisher least significant difference (LSD) test.

## Results

### Bacterial supernatants stimulated expression of immune response genes and impairs the osteogenic potential in PDLSC

**Fig 1:**
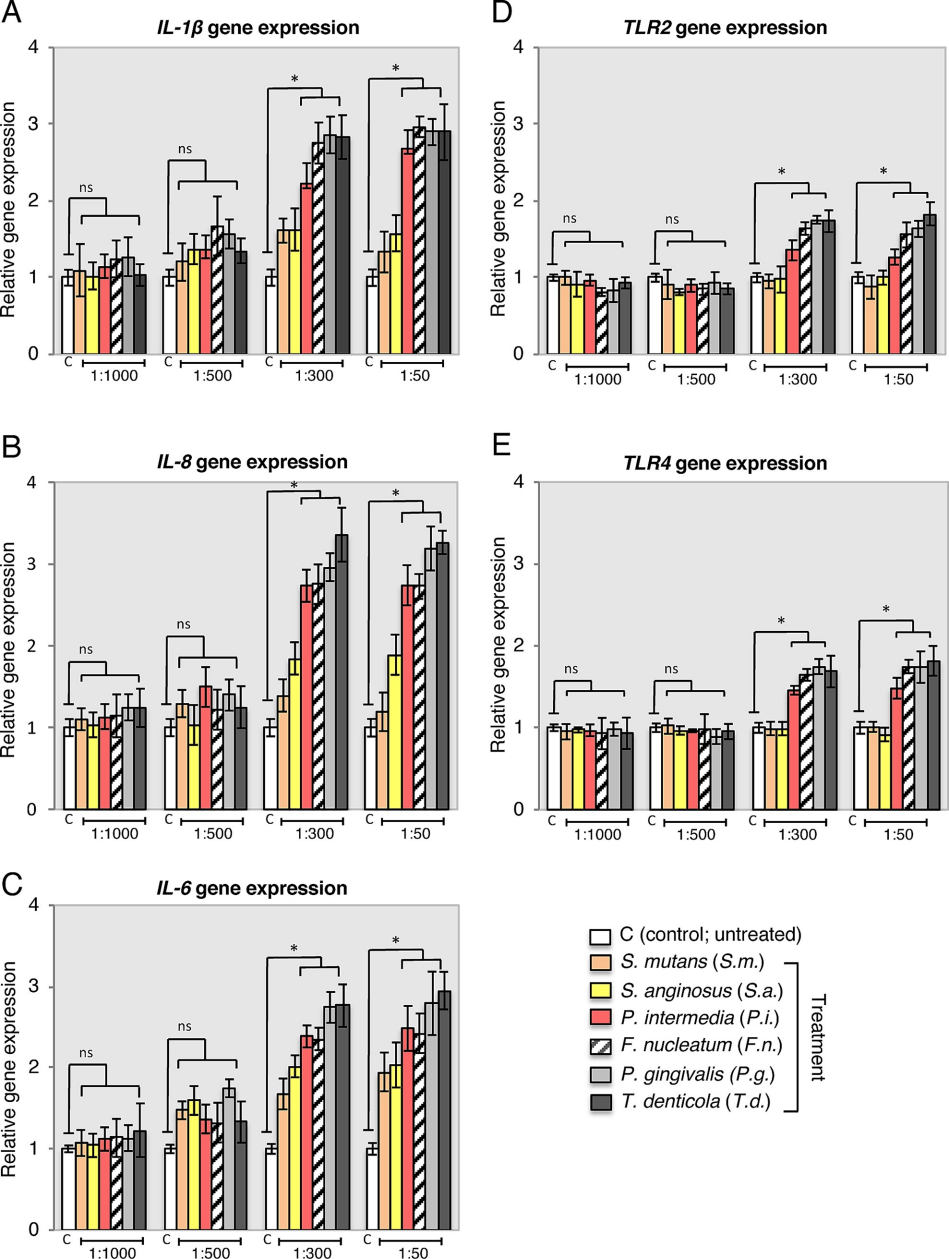
Bacterial supernatants modified expression of immune-related molecules in PDLSC. Gene expression levels determined by RT-PCR analysis showed upregulation of *IL-1β* (A) *IL-8* (B), *IL-6* (C), *TLR2* (D) and *TLR4* (E) in PDLSCs. Representative blots of at least three independent experiments are shown. Statistically significant differences presented as fold change relative to the untreated negative control (control, open columns) * p < 0.05. Mean ± S.D.

**Fig 2:**
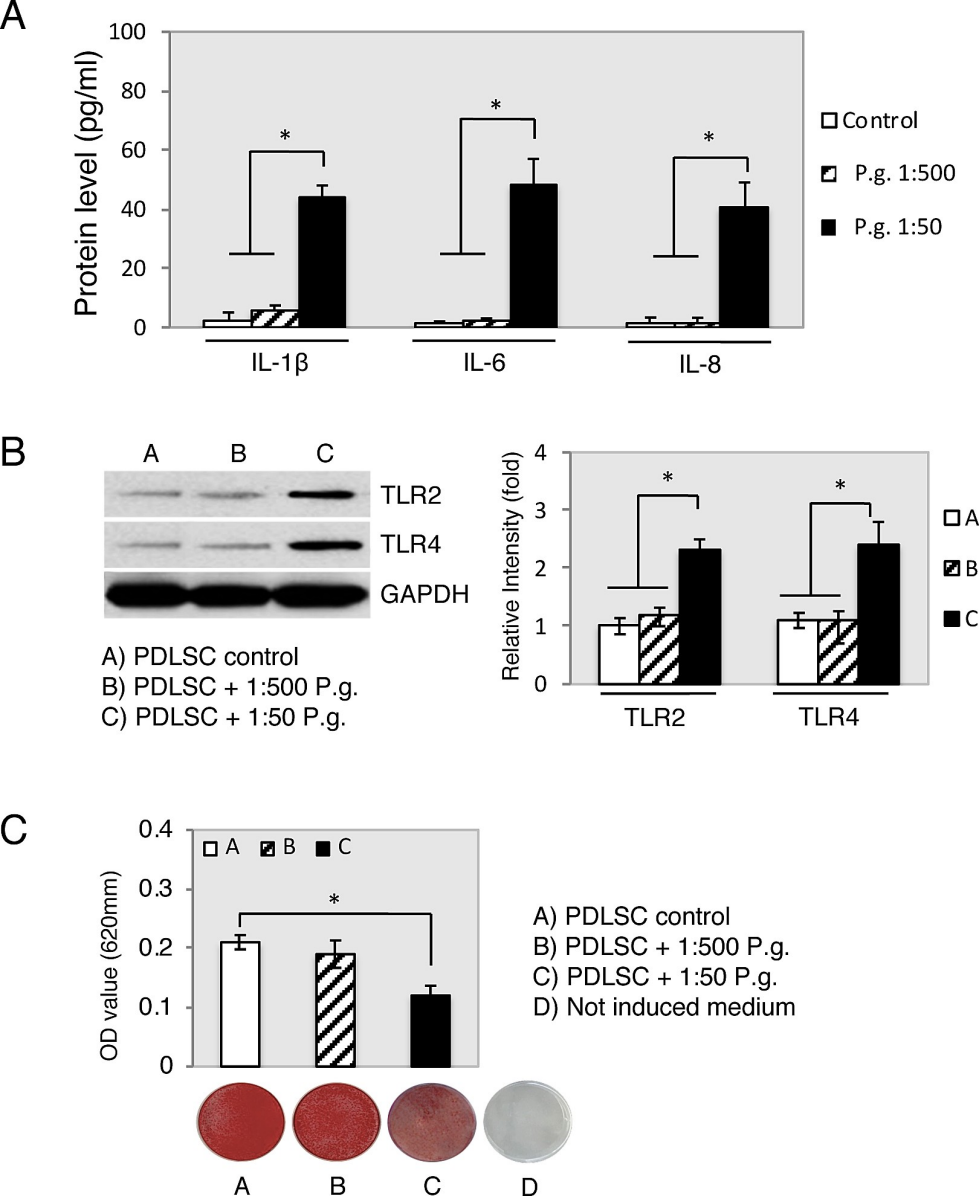
*P*. *gingivalis* supernatant impairs the osteogenic potential of PDLSC. (A) IL-1 β, IL-6 and IL-8 production was measured using ELISA after PDLSCs were treated with *P*. *gingivalis* (*P*.*g*.) supernatant’s dilutions (1:500 and 1:50), or untreated cells as control simultaneously for 24 h. IL-1 β, IL-6 and IL-8 is expressed in pg/ml ( ± standard deviation). (B) Western blot analysis showed the protein expression of TLR2, TLR4 and GAPDH was used as the internal control. (C) Confluent PDLSC were stained with Alizarin Red after 21 days of cultivation in osteogenic medium containing *P*. *gingivalis* supernatant’s dilutions (1:500 and 1:50) and Alizarin Red was then extracted and measured for light absorbance at 405 nm. *p < 0.05 as determined by unpaired two-tailed t tests. Data from three biologically independent replicates.

The effects of different dilutions (untreated, 1:1000, 1:500, 1:300 and 1:50) of bacterial supernatants (*S*. *mutans*, *S*. *anginosus*, *P*. *intermedia*, *F*. *nucleatum*, *P*. *gingivalis and T*. *denticola*) on the immunomodulatory related gene expression levels (*IL-1β*, *IL-6*, *IL-8*, *TLR2* and *TLR4*) in PDLSCs were investigated. RT-PCR analysis determined that 24 h treatment with 1:1000 and 1:500 of all bacterial species had no impact on constitutive gene expression in PDLSCs (Fig 1). Expression augmentation for *IL-6*, *IL-8* and *IL-1β* was found up to 3.5-fold higher when in cells exposed to 1:300 and 1:50 dilutions of supernatants derived specially from *P*. *intermedia*, *F*. *nucleatum*, *P*. *gingivalis* and *T*. *denticola* (Fig 1). Whereas no significant expression was observed for *S*. *mutans* and *S anginosus*. Expression of *TLR2* and *TLR4* was found to be maximum 2-fold compared to the control (Fig 1), also when in cells exposed to 1:300 and 1:50 dilutions of supernatants derived from *P*. *intermedia*, *F*. *nucleatum*, *P*. *gingivalis* and *T*. *denticola*. Values of PDLSC cultivated in absence of supernatants (control) were set as 1. * p < 0.05. Mean ± S.D. The statistical linear mixed models analysis confirmed the explorative and pairwise comparison results. In addition, PDLSCs culture supernatants were analyzed after 24 hours of treatment for IL-1β, IL-6 and IL-8 production using ELISA. A significant increase in IL-1β, IL-6 and IL-8 protein levels was detected with *P*. *gingivalis* 1:50 dilution treatment compared to *P*. *gingivalis* 1:500 dilution and the control (Fig 2, *p < 0.05). To ascertain the role of *P*. *gingivalis* bacterial supernatant in the activation of the TLR2 and TLR4 of PDLSCs, we stimulated PDLSCs by *P*. *gingivalis* bacterial supernatants (1:500 and 1:50) for 24 h. Western blot analysis showed that the expression of both TLR2 and TLR4 in PDLSCs was not altered by *P*. *gingivalis* 1:500 dilution treatment. However, *P*. *gingivalis* 1:50 dilution treatment increased TLR2 and TLR4 protein concentration in 2 folds compared to control (Fig 2, *p < 0.05). To verify the role of *P*. *gingivalis* bacterial supernatant in regulating the PDLSCs osteogenic differentiation, *P*. *gingivalis* supernatants (1:500 and 1:50) were added to the osteogenic induction medium. Alizarin red S staining showed that *P*. *gingivalis* supernatant 1:500 dilution impaired the osteogenic differentiation ability of PDLSC, which was demonstrated by the decreased formation number of calcified nodules stained with Alizarin Red compared with the control group after 21 days induction (Fig 2, day 21, *p < 0.05).

### Effect of *P*. *gingivalis* and *T*. *denticola* supernatants on PDLSC viability and proliferation

**Fig 3:**
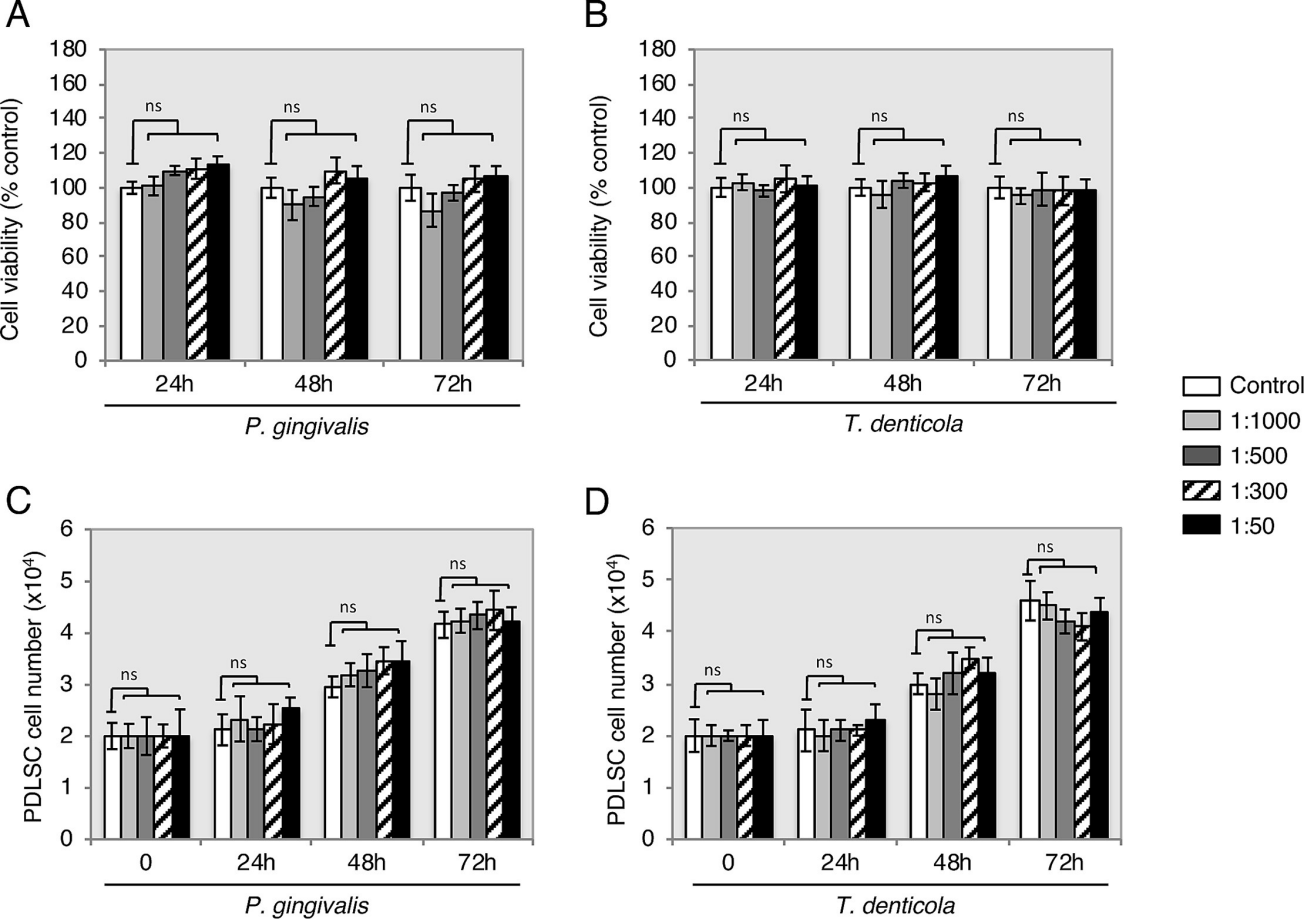
Viability and proliferation of PDLSC exposure to *P*. *gingivalis* and *T*. *denticola*. Upon supernatant treatment (untreated, 1:1000, 1:500, 1:300 and 1:50), PLDSC viability and proliferation percentages did not alter throughout periods of time (24 h, 48 h and 72 h) when compared with untreated controls (open columns). ns: non-significant. Data are shown as the mean ± S.D of 3 samples (3 wells each) from one representative experiment.

Cell viability and proliferation were performed using only *P*. *gingivalis* and *T*. *denticola* species in different dilutions (untreated, 1:1000, 1:500, 1:300 and 1:50). The results of MTT assay showed that all supernatants dilutions of *P*. *gingivalis* and *T*. *denticola* (Fig 3) had no significant effect on cell viability at 24 h, 48 h and 72 h compared with the control. The PDLSCs showed proliferation as expected by approximately doubling of the cell number throughout the observation periods of 24 h and 72 h. However, the proliferation rate compared to the controls within each individual period of time had no statistically significant alteration. Bacterial supernatants did not have an effect on proliferation in each time periods (Fig 3).

### Involvement of *P*. *gingivalis* and *T*. *denticola* supernatants in PDLSC migration

**Fig 4:**
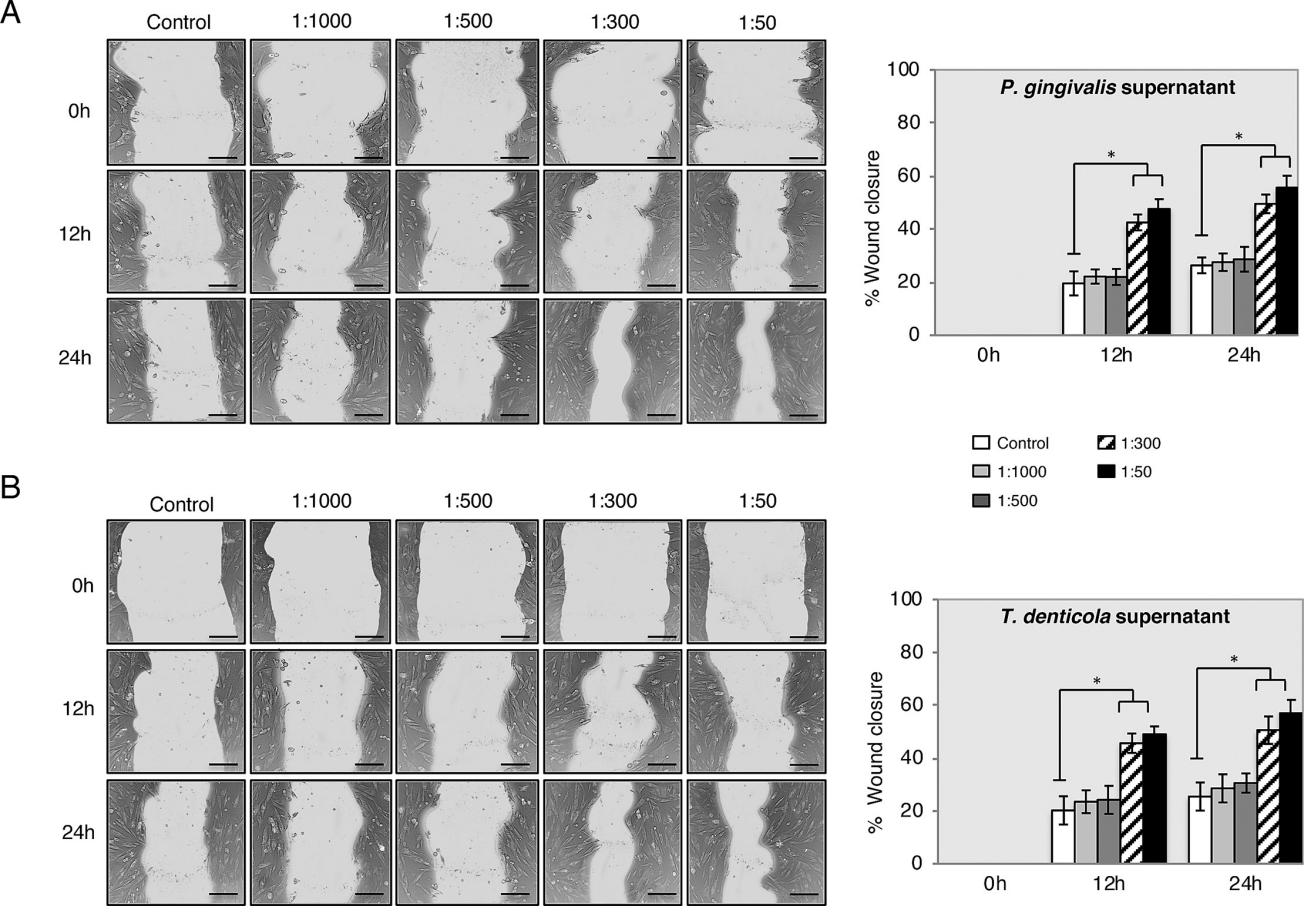
PDLSC migration was increased with supernatants of *P*. *gingivalis* and *T*. *denticola* by scratch wound healing assay. Representative images are shown from 3 independent experiments and light gray area define the areas lacking cells (Scale bar 120 μm). Images were analyzed using ImageJ software to calculate wound area. Data is expressed as the mean values of percentage wound closure relative to the corresponding 0 h time point and represent the mean percentage closure ± SEM (n = 3): *p < 0.01 vs. time-matched treated control for each time-point.

Following cell viability and proliferation, the migration capacity of PDLSC in the presence of *P*. *gingivalis* and *T*. *denticola* supernatants in different dilutions with the in vitro scratch wound healing assay was determined. Images were acquired at 0 h, 12 h and 24 h after exposing the cells to bacterial supernatants (Fig 4). After 24 h, PDLSC migrated and covered approximately 60% of the wound area observed at time zero when exposed to *P*. *gingivalis* and *T*. *denticola* at dilutions of 1:300 and 1:50.

### Transcriptional profile of PLDSC response to supernatants dilutions

**Fig 5:**
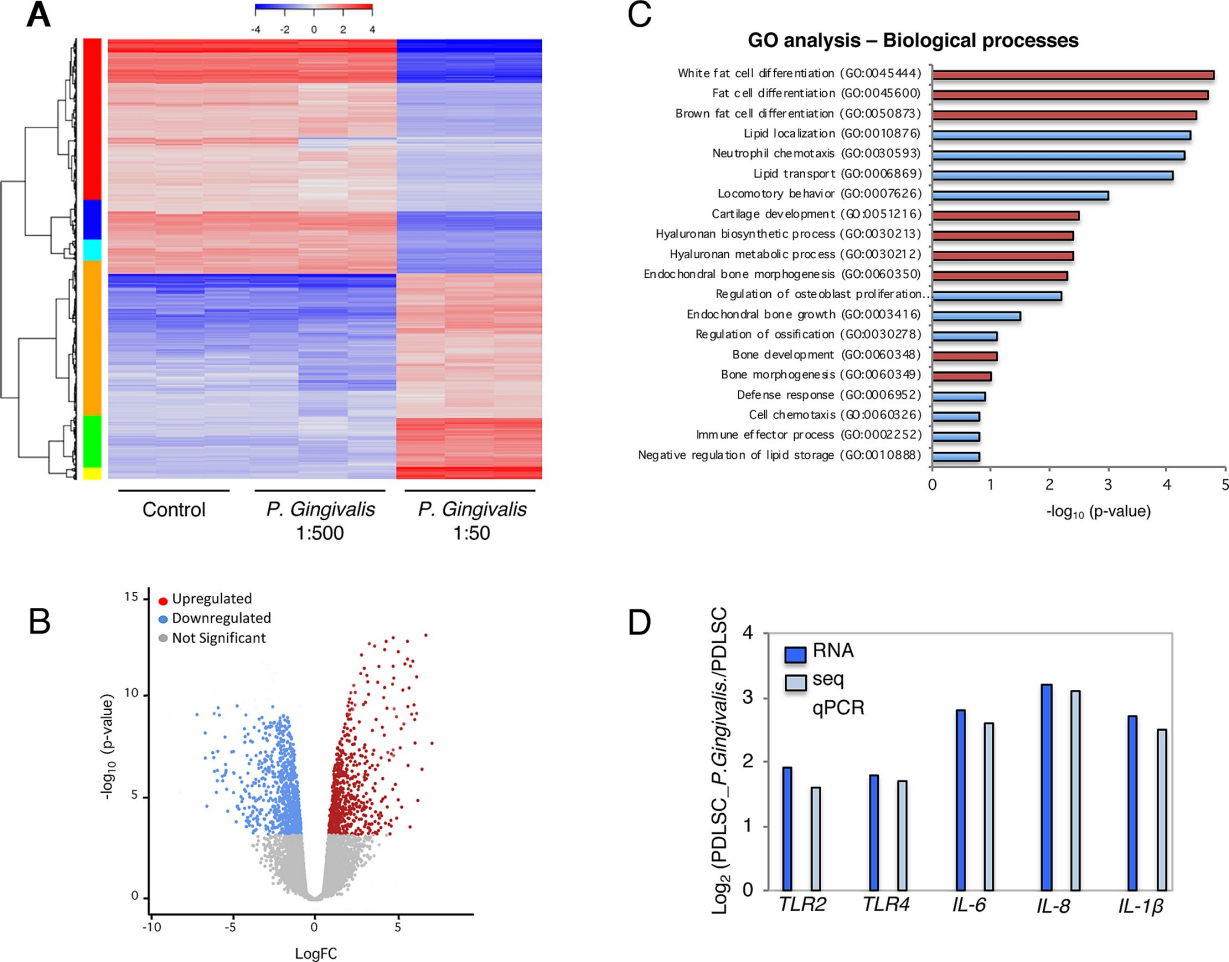
Transcriptomic analysis elucidates PDLSC response to bacterial supernatant-induced bioactivity. **(**A) Bacterial supernatant treated groups (1:500 and 1:50 dilutions) and untreated group (Ctrl) were marked. Heatmap showed significant gene profile for RNAseq data. Normalized log2 scale shown. (B) Volcano analysis was performed for significantly changed genes in the dilution of 1:50 supernatant treated group (red dots represented upregulated genes and blue dots represented downregulated genes, Fold change > 2.0, p < 0.05). (C) Significant GO terms of associated biological processes from differentially regulated genes from a ranking by enrichment scores (p < 0.05). Adipogenesis and chondrogenesis processes identified by red columns. Enrichment scores were calculated as −log_10_ (P-value), (D) Comparison between RNAseq and qPCR gene expression showed similar values.

Using the RNAseq method, the whole transcriptional profile of PLDSC in response to 1:500 and 1:50 dilutions of *P*. *gingivalis* supernatant (24 h cell exposure) was screened. All significantly changed genes were analyzed in heat map (Fig 5), which indicated more upregulated genes induced by *P*. *gingivalis* supernatant at 1:50 supernatant dilution. After RNAseq data processing, normalized and transformation, significantly changed genes of PLDSC with 1:50 dilution of *P*. *gingivalis* treatment compared to the untreated control were shown in the volcano Fig (Fold change > 2.0, p < 0.05 and False Discovery Rate < 0.05 were considered as significant) (Fig 5). The volcano analysis showed higher number of upregulated genes than downregulated genes. The total of 502 genes were found significantly changed after 1:50 *P*. *gingivalis* supernatant stimulation, of which 289 were upregulated, whereas 213 genes were downregulated. The analysis using KEGG pathway included 29 cytokines (10.9%), 32 signaling transducers (e.g NF-κB) genes (9.7%), 17 metabolism genes (6.1%), and 211 other functional genes (73.3%). To identify the key biological processes and pathways that are affected when the PDLSCs are treated with 1:50 dilution of *P*. *gingival* supernatant, gene ontology (GO) enrichment analysis performed by DAVID software allowed the listing of gene groups involved in the same biological processes. GO analyses of the common differentially expressed genes revealed that a large proportion of genes was involved in white and brown fat cell differentiation, lipid transport and lipid localization (Fig 5). PDLSCs 1:50-supernatant dilution treatment showed significant enrichment for 1,116 GO terms (p < 0.05), including 856 for biological processes. We then narrowed down key GO terms based on high significance (p value) to highlight the widespread effect of *P*. *gingivalis* supernatant on PDLSCs. The key GO terms significantly enriched in biological processes were white fat cell differentiation (GO: 0045444), white fat cell differentiation (GO: 0045444) and brown fat cell differentiation (GO: 0050873). The analysis also indicated positive regulation of cartilage development (GO:0051216), hyaluronan biosynthetic process (GO:0030213) and hyaluronan biosynthetic process (GO:0030213) (Fig 5). Terms related to biological process indicate strong biophysical interactions between PDLSCs and *P*. *gingivalis* supernatant. Also, in additional comparison, the expression of important genes participating in inflammation, which were significantly higher in PDLSCs treated with 1:50 dilution than in untreated controls was further validated by RT-PCR (Fig 5).

## Discussion

Considering the key role of PDLSC in periodontal tissue homeostasis and regeneration [10, 15, 16], the purpose of this study was to ascertain the effect of different periodontal bacterial supernatants on PDLSC immune response and cell differentiation potential. The results showed that periodontal bacteria supernatants could not only inhibit osteogenesis, but also increase the migration and immune response of PDLSC. In addition, this study also showed that these changes in biological process were most likely dependent upon increases in periodontal bacteria virulence.

Other previous in vitro studies using oral stem cells predominantly demonstrate the influence of LPS and fail to address the important effect of other virulent factors secreted or bacterial-derived molecules on a variety of cell responses. In this study, we sought to address the contributions made by bacterial-derived molecules other than just the role of LPS on stimulating PDLSCs inflammatory response and cell fate. Many pathogenicity determinants, which cause damage in a host organism, can be found in bacterial supernatants such as the ones employed in this study. For instance, several outer membrane proteins (lipd-A associated proteins/LAP) have been shown in *P*. *gingivalis* to be potent inducers of cytokine synthesis [22] with lowest concentration stimulation of only 10 ng/ml, whereas LPS from *P*. *gingivalis* is 1 log unit less active in this respect [22]. Several studies also have demonstrated the ability of fimbrial protein to stimulate cytokine synthesis in various cell types. *P*. *gingivalis* fimbriae (1 μg/ml) stimulated human gingival fibroblasts to release IL-1β [23] and P. intermedia (100 ng/ml) stimulated release of IL-1α, IL-1β, IL-6, and IL-8 [24]. Other virulent factors presents in supernatants are cell surface cysteine proteinases, such as *P*. *gingivalis* gingipains that can also be present in secreted soluble form on supernatants [25]. The gingipains have immunomodulatory multiple effects by enhancing cell-mediated immune response in oral tissues [26–29].

Many components of the supernatants collected for this study have increased cell response and may be associated to the virulence potency of the bacterial species. In previous animal studies on secreted determinants of pathogen virulence, the exopolysaccharide produced by *P*. *intermedia* and *P*. *gingivalis* was considered to be powerful menace for inflammation and abscess formation in mice [30]. Fimbriae and other microbial virulence proteins were also pointed to augment virulence in many pathogenic bacteria [23]. Moreover, microbial virulence proteins have evolved to interact with and possibly exploit TLRs and the cell pattern recognition system, in ways that increase bacterial pathogenicity [31]. *T*. *denticola* membrane-associated lipoproteins/lipooligosaccharides were also found to induce production of inflammatory mediators by mouse macrophages in a dose-dependent manner [32]. We believe that all those pathogenicity determinants from bacterial species used in this study may have played an important role on intensifying the observed PDLSCs response.

The literature suggests that pathogenic bacterial endotoxin concentrations in the subgingival biofilm vary greatly depending on the local micro-environmental conditions [33]. Following those reports, we decided to use bacterial supernatant dilutions, which could resemble the endotoxins concentrations likely to be found in the subgingival plaque of periodontal pockets. In those studies, for the single stimulation experiments as performed here, the bacterial supernatants were also directly diluted in the cell culture medium [20, 21]. The bacterial strains chosen for this study represent some important members of the oral biofilm. *P*. *intermedia*, *F*. *nucleatum*, *P*. *gingivalis* and *T*. *denticola* were used as species representative of the pathogenic subgingival biofilm related with periodontitis. *S*. *mutans* and *S*. *anginosus* were used as controls, to define the specificity of the results regarding the pathogenicity of the other gram-negative bacterial species.

Changes in PDLSCs lineage fate, caused by bacterial endotoxins, were observed in recent studies, which implicate bacterial endotoxins as inhibitors of PDLSCs osteogenic differentiation [11, 12, 34]. Accordingly, the transcriptional profile results presented here suggest that inflammation may be associated with inhibition of PDLSCs osteogenic capability. In comparison to literature assessing the effect of LPS on PDLSCs, adipogenesis and chondrogenesis in bacterial *P*. *gingivalis* supernatant treated PDLSCs observed in the current study also presented increased levels [35, 36]. Even though adipogenesis and chondrogenesis differentiation of PDLSCs have been questioned in vivo [37], bacterial endotoxins are still a pivot for change in PDLSCs differentiation into undesirable patterns of periodontal ligament tissue maintenance; directions associated with some additional pathologic changes. For example, the small size increments of neutral lipid droplets in cytosol correlation to various metabolic changes in cells, which appears during cellular senescence process and LPS-treated dental pulp stem cells oxidative stress [38, 39]. It may be speculated that PDLSC adipogenic differentiation is related to in vitro cell culture conditions and changes in energy metabolism. In addition, an inflammatory microenvironment may modulate immunomodulatory PDLSC properties [13, 14], which supports the findings of the current study where those inflammatory cytokines (*IL-6*, *IL-8*, *IL1 β*) and transmembrane cell-surface receptors (*TLR2* and *TLR4*) were significantly enhanced (Figs 1 and 2). This was further confirmed by the whole transcriptional profile RNAseq analysis. The increase in gene expression was dependent on the supernatant dilution and the bacterial virulence, as the expression was higher in cells exposed to higher dilutions of supernatants (i.e. 1:300–1:50) derived from strongly pathogenic bacteria (*P*. *intermedia*, *F*. *nucleatum*, *P*. *gingivalis* and *T*. *denticola*). These results may also support the hypothesis of the immune cell like function of periodontal ligament cells [37]. In contrast, the RT-PCR analysis showed lower augmentation of *TLR2* and *TLR4* expression under the influence of bacteria supernatant treatment. Possible explanations of this lower-expression could be due to cells adaptation mechanism to bacteria endotoxins exposure, or that TLR2 mostly identifies lipo-proteins, lipo-peptides or other virulence factors rather than LPS, whereas TLR4 recognizes LPS [39]. In fact, the family of the Toll-like receptors is involved in recognition of bacterial cell wall components [40]. Moreover, the treatment of PDLSC with bacterial supernatant of *P*. *gingivalis* and *T*. *denticola* did not increase cell viability and proliferation compared to the controls within each individual period of time analyzed (Fig 3), although previous studies suggested that migratory properties of stem cells enhanced by LPS treatment on murine odontoblasts-like cells, dental follicle periodontal cells, bone marrow mesenchymal stem cells [41, 42] and periodontal ligament cells [43, 44]. In contrast to the proliferation results, PDLSC presented significantly higher motility activity when challenged with *P*. *gingivalis* and *T*. *denticola* at 1:300 and 1:50 dilutions (Fig 4), whereas dilutions of 1:1000 and 1:500 did not elicit migration. The unchanged migration upon lower supernatant dilution treatment could be explained by lower concentration of endotoxins content, which reduces supernatant stimulation potency. And it indicates that there is possibly a threshold for PDLSCs responsiveness to bacterial endotoxins. However, the data obtained from dilutions of 1:300 and 1:500 supernatant stimulation verifies the stimulatory impact of *P*. *gingivalis* and *T*. *denticola* bacterial endotoxins on PDLSCs mobility, which could play a crucial role in the periodontal tissue repair process. This theory was also confirmed in previous studies which showed that LPS stimulated contractility of PDLSCs with elevated myofibroblast marker expression of α-SMA, TGF-β, and FN proteins [34, 45] specially found in periodontal ligament fibroblasts in response to chemical stress signals [46].

This study has provided further evidence of the broader role played by different periodontal bacterial species as to their influence on PDLSCs cell fate and differentiation properties. Also, the increase in gene expression related to adipogenic or chondrogenic noted in the RNA sequencing (Fig 5) and inhibition on osteogenesis (Fig 2) in this study suggests the negative effect of bacterial endotoxins on periodontal regeneration. For instance, by TLR4 mediation, LPS was found to activate the host cells in the periodontium, including polymorphonuclear leukocytes, macrophages, fibroblasts and the epithelium [47, 48]. And inhibition of TLR4 or NF-κB pathway impaired alveolar bone loss caused by LPS treatment [12]. Nevertheless, the detailed mechanism still needs further investigation, as the mechanism of LPS induced periodontitis is complicated and not completely understood. Aside from endotoxin modification of PDLSC differentiation, LPS can induce alveolar bone loss by stimulating the secretion of proinflammatory cytokines (IL-1β, TNF-α or IL-6). LPS can also induce the secretion of matrix metalloproteinases, formation of osteoclasts stimulation, causing direct damage to periodontal tissues [20]. An important question in cellular susceptibility to inflammation is to what extent the variability in pathogen species could induce higher or lower immune response. Strains of bacterial species are not equally pathogenic due to dissimilarity in virulence factor expression. Following this idea, the contribution of the pathogenic bacteria on the cytokine production was shown in our previous study [19], in which the same in vitro methodology used in the present study enabled the determination of cell-type specific response to different periodontal bacterial species according to its pathogenicity. Nevertheless, the immunomodulatory activity of PDLSC still need to be assessed by additional studies to clarify which particles other that LPS can govern inflammation or whether it is mediated through suppression of other downstream signaling pathways.

In summary, this study demonstrates that different bacterial stimulation induces immunoresponse, cell motility and most importantly the differentiation potential of PDLSC. Stem cell response to periodontal bacteria raises questions on the multipotent cells contribution to both outbreak of inflammation and periodontal tissue regeneration. Immunomodulation of stem cells through bacterial stimulation is a matter of great interest, as it may allow the development of therapeutic interventions for inflammatory diseases such as periodontitis.

## Supporting information

S1 Fig Growth curves of different bacteria species at the optical density measurements. Correspondence was established between OD 550 nm measured from 0 to 20h and bacteria counted in microscopy. Colonies of *S*. *mutans*, *S*. *anginosus*, *P*. *intermedia*, *F*. *nucleatum*, *P*. *gingivalis* and *T*. *denticola* were cultured under anaerobically conditions (6 h, 37°C, 10% CO_2_) in 5 mL brain heart infusion broth (Merck, Darmstadt, Germany). Cells from the logarithmic growth phase were used for the study after overnight standardization of bacterial cultures in 5 mL PBS to achieve a bacterial cell concentration of 10^6^ CFU/mL. The density of the resulting inoculum was measured using a MicroSpeak densitometer and confirmed by single colony counting after 24-hour growth on brain heart infusion broth medium under the above-specified conditions. The number of grown bacterial colonies (CFU/mL) was calculated. The results are means of three experiments and error bars represent standard deviations determined from at least three replicates.
(TIF)

S1 File Minimal data set. (DOCX)
