# Supplementary figures and images for: Periodontal bacterial supernatants modify differentiation, migration and inflammatory cytokine expression in human periodontal ligament stem cells
Source: PLoS One. 2019 Jul 3;14(7):e0219181. doi: 10.1371/journal.pone.0219181 (PMC6609032; doi:10.1371/journal.pone.0219181)

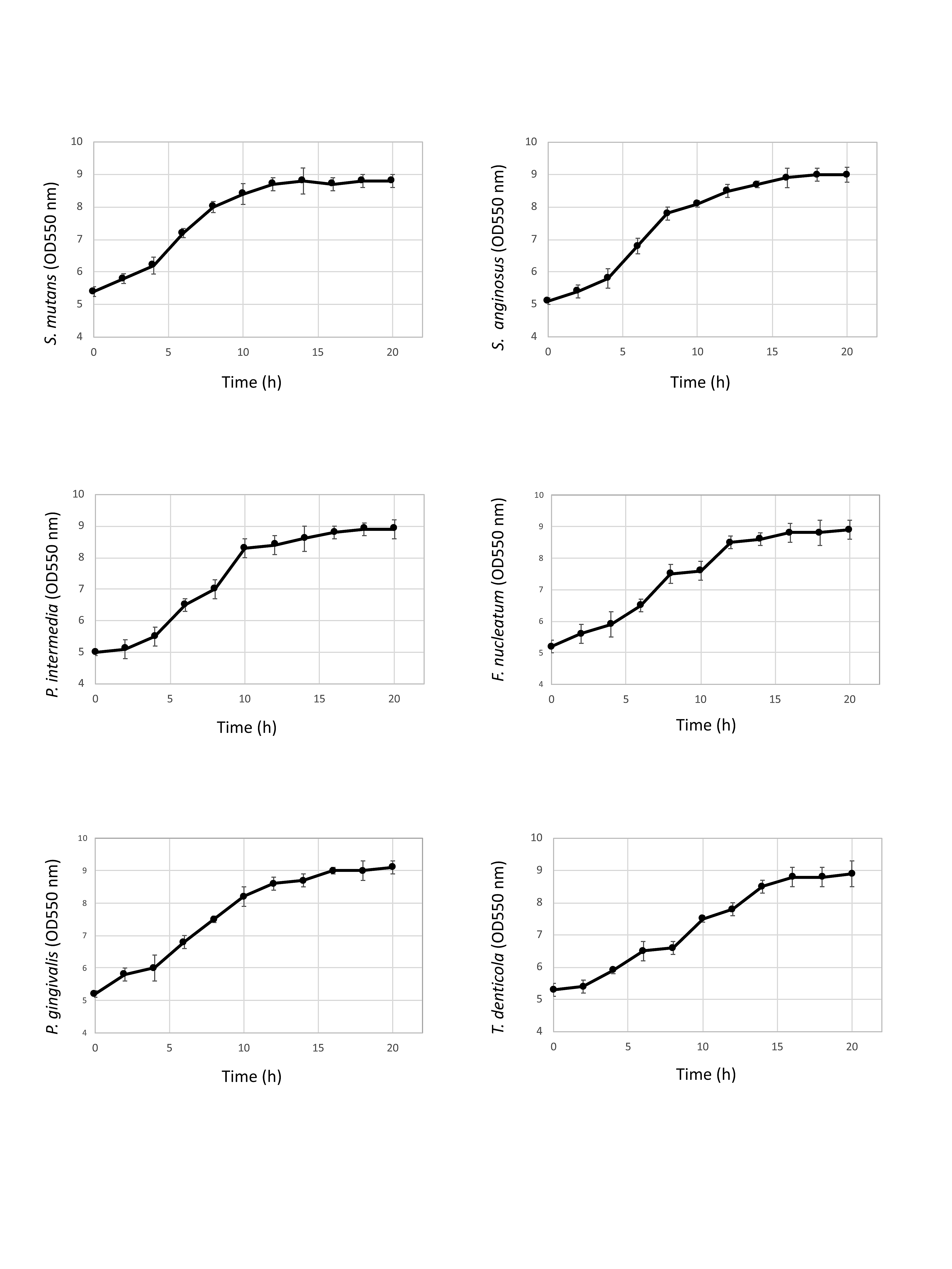

Supplement: S1 Fig — Correspondence was established between OD 550 nm measured from 0 to 20h and bacteria counted in microscopy. Colonies of S. mutans, S. anginosus, P. intermedia, F. nucleatum, P. gingivalis and T. denticola were cultured under anaerobically conditions (6 h, 37°C, 10% CO2) in 5 mL brain heart infusion broth (Merck, Darmstadt, Germany). Cells from the logarithmic growth phase were used for the study after overnight standardization of bacterial cultures in 5 mL PBS to achieve a bacterial cell concentration of 106 CFU/mL. The density of the resulting inoculum was measured using a MicroSpeak densitometer and confirmed by single colony counting after 24-hour growth on brain heart infusion broth medium under the above-specified conditions. The number of grown bacterial colonies (CFU/mL) was calculated. The results are means of three experiments and error bars represent standard deviations determined from at least three replicates. (TIF) [file pone.0219181.s001.tif]
