# Supplementary material for: Periodontal bacterial supernatants modify differentiation, migration and inflammatory cytokine expression in human periodontal ligament stem cells
Source: PLoS One. 2019 Jul 3;14(7):e0219181. doi: 10.1371/journal.pone.0219181 (PMC6609032; doi:10.1371/journal.pone.0219181)
Supplement: S1 File — (DOCX) [file pone.0219181.s002.docx]

| **Figure #** | **Mean** | **S.D** | **Statistical method used** | **P value** | **# samples** |
| --- | --- | --- | --- | --- | --- |
| **Fig. 1A** |  |  | Linear mixed model statistics for large series of pairwise comparisons and paired-tests | * P <0.05 |  |
| Control untreated | 1 | 0.1041 |  |  | 9 |
| 100pg/ml *S.m.* | 1.0974 | 0.3421 |  |  | 9 |
| 100pg/ml *S.a.* | 1.0231 | 0.1774 |  |  | 9 |
| 100pg/ml *P.i.* | 1.1274 | 0.1851 |  |  | 9 |
| 100pg/ml *F.n.* | 1.2441 | 0.2412 |  |  | 9 |
| 100pg/ml *P.g.* | 1.2737 | 0.2534 |  |  | 9 |
| 100pg/ml *T.d.* | 1.0344 | 0.1423 |  |  | 9 |
| Control untreated | 1 | 0.1052 |  |  | 9 |
| 50ng/ml *S.m.* | 1.2031 | 0.2432 |  |  | 9 |
| 50ng/ml *S.a.* | 1.3742 | 0.1975 |  |  | 9 |
| 50ng/ml *P.i.* | 1.3535 | 0.1852 |  |  | 9 |
| 50ng/ml *F.n.* | 1.6741 | 0.3854 |  |  | 9 |
| 50ng/ml *P.g.* | 1.5696 | 0.1914 |  |  | 9 |
| 50ng/ml *T.d.* | 1.3577 | 0.1622 |  |  | 9 |
| Control untreated | 1 | 0.1066 |  |  | 9 |
| 200ng/ml *S.m.* | 1.6132 | 0.1565 |  |  | 9 |
| 200ng/ml *S.a.* | 1.6657 | 0.2758 |  |  | 9 |
| 200ng/ml *P.i.* | 2.2177 | 0.2752 |  |  | 9 |
| 200ng/ml *F.n.* | 2.7567 | 0.2674 |  |  | 9 |
| 200ng/ml *P.g.* | 2.8633 | 0.2392 |  |  | 9 |
| 200ng/ml *T.d.* | 2.8337 | 0.2851 |  |  | 9 |
| Control untreated | 1 | 0.1052 |  |  | 9 |
| 500ng/ml *S.m.* | 1.3341 | 0.2656 |  |  | 9 |
| 500ng/ml *S.a.* | 1.5795 | 0.2356 |  |  | 9 |
| 500ng/ml *P.i.* | 2.6874 | 0.2855 |  |  | 9 |
| 500ng/ml *F.n.* | 2.9674 | 0.1352 |  |  | 9 |
| 500ng/ml *P.g.* | 2.8977 | 0.1712 |  |  | 9 |
| 500ng/ml *T.d.* | 2.8975 | 0.3641 |  |  | 9 |
| **Fig. 1B** |  |  | Linear mixed model statistics for large series of pairwise comparisons and paired-tests | *P <0.05 |  |
| Control untreated | 1 | 0.1012 |  |  | 9 |
| 100pg/ml *S.m.* | 1.1032 | 0.1342 |  |  | 9 |
| 100pg/ml *S.a.* | 1.0342 | 0.1527 |  |  | 9 |
| 100pg/ml *P.i.* | 1.1354 | 0.1652 |  |  | 9 |
| 100pg/ml *F.n.* | 1.1444 | 0.2624 |  |  | 9 |
| 100pg/ml *P.g.* | 1.2494 | 0.1611 |  |  | 9 |
| 100pg/ml *T.d.* | 1.2422 | 0.2347 |  |  | 9 |
| Control untreated | 1 | 0.1022 |  |  | 9 |
| 50ng/ml *S.m.* | 1.2946 | 0.1675 |  |  | 9 |
| 50ng/ml *S.a.* | 1.0334 | 0.2443 |  |  | 9 |
| 50ng/ml *P.i.* | 1.4977 | 0.2185 |  |  | 9 |
| 50ng/ml *F.n.* | 1.2215 | 0.2441 |  |  | 9 |
| 50ng/ml *P.g.* | 1.3994 | 0.1937 |  |  | 9 |
| 50ng/ml *T.d.* | 1.2532 | 0.2574 |  |  | 9 |
| Control untreated | 1 | 0.1104 |  |  | 9 |
| 200ng/ml *S.m.* | 1.3937 | 0.1974 |  |  | 9 |
| 200ng/ml *S.a.* | 1.8473 | 0.1971 |  |  | 9 |
| 200ng/ml *P.i.* | 2.7352 | 0.1375 |  |  | 9 |
| 200ng/ml *F.n.* | 2.7537 | 0.2422 |  |  | 9 |
| 200ng/ml *P.g.* | 2.9624 | 0.1715 |  |  | 9 |
| 200ng/ml *T.d.* | 3.3595 | 0.3347 |  |  | 9 |
| Control untreated | 1 | 0.1087 |  |  | 9 |
| 500ng/ml *S.m.* | 1.1929 | 0.2356 |  |  | 9 |
| 500ng/ml *S.a.* | 1.8906 | 0.2457 |  |  | 9 |
| 500ng/ml *P.i.* | 2.7418 | 0.2885 |  |  | 9 |
| 500ng/ml *F.n.* | 2.7271 | 0.1524 |  |  | 9 |
| 500ng/ml *P.g.* | 3.1916 | 0.2694 |  |  | 9 |
| 500ng/ml *T.d.* | 3.2657 | 0.1427 |  |  | 9 |
| **Fig. 1C** |  |  | Linear mixed model statistics for large series of pairwise comparisons and paired-tests | *P <0.05 |  |
| Control untreated | 1 | 0.0413 |  |  | 9 |
| 100pg/ml *S.m.* | 1.0734 | 0.2185 |  |  | 9 |
| 100pg/ml *S.a.* | 1.0432 | 0.1442 |  |  | 9 |
| 100pg/ml *P.i.* | 1.1213 | 0.1524 |  |  | 9 |
| 100pg/ml *F.n.* | 1.1355 | 0.2347 |  |  | 9 |
| 100pg/ml *P.g.* | 1.1324 | 0.1641 |  |  | 9 |
| 100pg/ml *T.d.* | 1.2321 | 0.3323 |  |  | 9 |
| Control untreated | 1 | 0.0523 |  |  | 9 |
| 50ng/ml *S.m.* | 1.4754 | 0.1674 |  |  | 9 |
| 50ng/ml *S.a.* | 1.5964 | 0.1774 |  |  | 9 |
| 50ng/ml *P.i.* | 1.3689 | 0.1854 |  |  | 9 |
| 50ng/ml *F.n.* | 1.3244 | 0.2441 |  |  | 9 |
| 50ng/ml *P.g.* | 1.7467 | 0.1174 |  |  | 9 |
| 50ng/ml *T.d.* | 1.3322 | 0.2541 |  |  | 9 |
| Control untreated | 1 | 0.0613 |  |  | 9 |
| 200ng/ml *S.m.* | 1.6764 | 0.1124 |  |  | 9 |
| 200ng/ml *S.a.* | 2.0173 | 0.1352 |  |  | 9 |
| 200ng/ml *P.i.* | 2.3861 | 0.1452 |  |  | 9 |
| 200ng/ml *F.n.* | 2.3552 | 0.1377 |  |  | 9 |
| 200ng/ml *P.g.* | 2.7431 | 0.1985 |  |  | 9 |
| 200ng/ml *T.d.* | 2.7654 | 0.2621 |  |  | 9 |
| Control untreated | 1 | 0.0723 |  |  | 9 |
| 500ng/ml *S.m.* | 1.9475 | 0.1917 |  |  | 9 |
| 500ng/ml *S.a.* | 2.0334 | 0.2767 |  |  | 9 |
| 500ng/ml *P.i.* | 2.4839 | 0.2854 |  |  | 9 |
| 500ng/ml *F.n.* | 2.4244 | 0.2441 |  |  | 9 |
| 500ng/ml *P.g.* | 2.7927 | 0.3917 |  |  | 9 |
| 500ng/ml *T.d.* | 2.9514 | 0.2329 |  |  | 9 |
| **Fig. 1D** |  |  | Linear mixed model statistics for large series of pairwise comparisons and paired-tests | *P <0.05 |  |
| Control untreated | 1 | 0.0413 |  |  | 9 |
| 100pg/ml *S.m.* | 0.9981 | 0.0913 |  |  | 9 |
| 100pg/ml *S.a.* | 0.9140 | 0.1617 |  |  | 9 |
| 100pg/ml *P.i.* | 0.9676 | 0.0724 |  |  | 9 |
| 100pg/ml *F.n.* | 0.8124 | 0.0524 |  |  | 9 |
| 100pg/ml *P.g.* | 0.8324 | 0.1516 |  |  | 9 |
| 100pg/ml *T.d.* | 0.9320 | 0.0723 |  |  | 9 |
| Control untreated | 1 | 0.0583 |  |  | 9 |
| 50ng/ml *S.m.* | 0.9123 | 0.1911 |  |  | 9 |
| 50ng/ml *S.a.* | 0.8137 | 0.0414 |  |  | 9 |
| 50ng/ml *P.i.* | 0.8971 | 0.0817 |  |  | 9 |
| 50ng/ml *F.n.* | 0.8479 | 0.0723 |  |  | 9 |
| 50ng/ml *P.g.* | 0.9302 | 0.1411 |  |  | 9 |
| 50ng/ml *T.d.* | 0.8535 | 0.0725 |  |  | 9 |
| Control untreated | 1 | 0.0613 |  |  | 9 |
| 200ng/ml *S.m.* | 0.9518 | 0.0982 |  |  | 9 |
| 200ng/ml *S.a.* | 0.9764 | 0.1714 |  |  | 9 |
| 200ng/ml *P.i.* | 1.3562 | 0.1313 |  |  | 9 |
| 200ng/ml *F.n.* | 1.6415 | 0.0824 |  |  | 9 |
| 200ng/ml *P.g.* | 1.7548 | 0.0519 |  |  | 9 |
| 200ng/ml *T.d.* | 1.7359 | 0.1424 |  |  | 9 |
| Control untreated | 1 | 0.0723 |  |  | 9 |
| 500ng/ml *S.m.* | 0.8765 | 0.1519 |  |  | 9 |
| 500ng/ml *S.a.* | 0.9299 | 0.0923 |  |  | 9 |
| 500ng/ml *P.i.* | 1.2697 | 0.1002 |  |  | 9 |
| 500ng/ml *F.n.* | 1.5582 | 0.1625 |  |  | 9 |
| 500ng/ml *P.g.* | 1.6351 | 0.1062 |  |  | 9 |
| 500ng/ml *T.d.* | 1.8326 | 0.1523 |  |  | 9 |
| **Fig. 1E** |  |  | Linear mixed model statistics for large series of pairwise comparisons and paired-tests | *P <0.05 |  |
| Control untreated | 1 | 0.0522 |  |  | 9 |
| 100pg/ml *S.m.* | 0.9518 | 0.0937 |  |  | 9 |
| 100pg/ml *S.a.* | 1.0108 | 0.0324 |  |  | 9 |
| 100pg/ml *P.i.* | 0.9697 | 0.0718 |  |  | 9 |
| 100pg/ml *F.n.* | 1.4833 | 0.1914 |  |  | 9 |
| 100pg/ml *P.g.* | 0.9832 | 0.0816 |  |  | 9 |
| 100pg/ml *T.d.* | 0.9320 | 0.1913 |  |  | 9 |
| Control untreated | 1 | 0.0621 |  |  | 9 |
| 50ng/ml *S.m.* | 1.0196 | 0.0934 |  |  | 9 |
| 50ng/ml *S.a.* | 0.9676 | 0.0514 |  |  | 9 |
| 50ng/ml *P.i.* | 0.9641 | 0.0177 |  |  | 9 |
| 50ng/ml *F.n.* | 0.9848 | 0.1825 |  |  | 9 |
| 50ng/ml *P.g.* | 0.8930 | 0.0919 |  |  | 9 |
| 50ng/ml *T.d.* | 0.9535 | 0.0934 |  |  | 9 |
| Control untreated | 1 | 0.0911 |  |  | 9 |
| 200ng/ml *S.m.* | 1.0196 | 0.0827 |  |  | 9 |
| 200ng/ml *S.a.* | 0.9676 | 0.0819 |  |  | 9 |
| 200ng/ml *P.i.* | 1.4582 | 0.0535 |  |  | 9 |
| 200ng/ml *F.n.* | 1.6452 | 0.0727 |  |  | 9 |
| 200ng/ml *P.g.* | 1.7481 | 0.0917 |  |  | 9 |
| 200ng/ml *T.d.* | 1.6865 | 0.1925 |  |  | 9 |
| Control untreated | 1 | 0.0511 |  |  | 9 |
| 500ng/ml *S.m.* | 1.0108 | 0.0623 |  |  | 9 |
| 500ng/ml *S.a.* | 0.9186 | 0.0834 |  |  | 9 |
| 500ng/ml *P.i.* | 1.4832 | 0.1275 |  |  | 9 |
| 500ng/ml *F.n.* | 1.7493 | 0.0823 |  |  | 9 |
| 500ng/ml *P.g.* | 1.7205 | 0.1926 |  |  | 9 |
| 500ng/ml *T.d.* | 1.8172 | 0.1813 |  |  | 9 |
| **Fig. 2A** |  |  | ANOVA/post hoc Fisher LSD test: compare selected pairs | *P <0.05 |  |
| Control untreated | 2.1231 | 3.1213 |  |  | 6 |
| 50ng/ml *P.g.* ILb1 | 5.4123 | 2.0633 |  |  | 6 |
| 500ng/ml *P.g.* ILb1 | 44.213 | 4.1251 |  |  | 6 |
| Control untreated | 1.0524 | 1.0252 |  |  | 6 |
| 50ng/ml *P.g.* IL6 | 2.0521 | 1.0855 |  |  | 6 |
| 500ng/ml *P.g.* IL6 | 48.521 | 9.1255 |  |  | 6 |
| Control untreated | 1.3251 | 2.0552 |  |  | 6 |
| 50ng/ml *P.g.* IL8 | 1.7213 | 1.5242 |  |  | 6 |
| 500ng/ml *P.g.* IL8 | 41.285 | 8.0252 |  |  | 6 |
| **Fig. 2B** |  |  | ANOVA/post hoc Fisher LSD test: compare selected pairs | *P <0.05 |  |
| PDLSC control | 1.0411 | 0.1185 |  |  | 6 |
| 50ng/ml *P.g.* TLR2 | 1.2351 | 0.1253 |  |  | 6 |
| 500ng/ml *P.g.* TLR2 | 2.3251 | 0.2521 |  |  | 6 |
| PDLSC control | 1.1143 | 0.1325 |  |  | 6 |
| 50ng/ml *P.g.* TLR4 | 1.1225 | 0.1652 |  |  | 6 |
| 500ng/ml *P.g.* TLR4 | 2.4213 | 0.4223 |  |  | 6 |
| **Fig. 2C** |  |  | ANOVA/post hoc Fisher LSD test: compare selected pairs | *P <0.05 |  |
| PDLSC control | 0.2152 | 0.0123 |  |  | 6 |
| PDLSC 50ng/ml*P.g.* | 0.1923 | 0.0232 |  |  | 6 |
| PDLSC 500ng/ml*P.g.* | 0.1252 | 0.0165 |  |  | 6 |
| **Fig. 3A** | **(%)** |  | ANOVA/post hoc Fisher LSD test: compare selected pairs | *P <0.05 |  |
| 24h Control untreated*P.g.* | 100 | 3.5212 |  |  | 9 |
| 24h 100pg/ml *P.g.* | 101 | 5.4247 |  |  | 9 |
| 24h 50ng/ml *P.g.* | 110 | 2.7274 |  |  | 9 |
| 24h 200ng/ml *P.g.* | 111 | 5.9174 |  |  | 9 |
| 24h 500ng/ml *P.g.* | 114 | 4.2347 |  |  | 9 |
| 48h Control untreated*P.g.* | 100 | 5.8714 |  |  | 9 |
| 48h 100pg/ml *P.g.* | 90 | 8.7114 |  |  | 9 |
| 48h 50ng/ml *P.g.* | 95 | 5.5871 |  |  | 9 |
| 48h 200ng/ml *P.g.* | 110 | 7.5839 |  |  | 9 |
| 48h 500ng/ml *P.g.* | 105 | 7.5824 |  |  | 9 |
| 72h Control untreated*P.g.* | 100 | 7.5378 |  |  | 9 |
| 72h 100pg/ml *P.g.* | 87 | 9.6745 |  |  | 9 |
| 72h 50ng/ml *P.g.* | 97 | 4.5852 |  |  | 9 |
| 72h 200ng/ml *P.g.* | 105 | 7.5415 |  |  | 9 |
| 72h 500ng/ml *P.g.* | 106 | 6.5852 |  |  | 9 |
| **Fig. 3B** | **(%)** |  | ANOVA/post hoc Fisher LSD test: compare selected pairs | *P <0.05 |  |
| 24h Control untreated*T.d.* | 100 | 5.5123 |  |  | 9 |
| 24h 100pg/ml *T.d.* | 103 | 4.6141 |  |  | 9 |
| 24h 50ng/ml *T.d.* | 98 | 3.3254 |  |  | 9 |
| 24h 200ng/ml *T.d.* | 105 | 7.8255 |  |  | 9 |
| 24h 500ng/ml *T.d.* | 101 | 5.5241 |  |  | 9 |
| 48h Control untreated*T.d.* | 100 | 4.8213 |  |  | 9 |
| 48h 100pg/ml *T.d.* | 96 | 7.8255 |  |  | 9 |
| 48h 50ng/ml *T.d.* | 104 | 4.3852 |  |  | 9 |
| 48h 200ng/ml *T.d.* | 103 | 5.1241 |  |  | 9 |
| 48h 500ng/ml *T.d.* | 106 | 6.6524 |  |  | 9 |
| 72h Control untreated*T.d.* | 100 | 6.3213 |  |  | 9 |
| 72h 100pg/ml *T.d.* | 95 | 4.6374 |  |  | 9 |
| 72h 50ng/ml *T.d.* | 99 | 9.5824 |  |  | 9 |
| 72h 200ng/ml *T.d.* | 98 | 8.2524 |  |  | 9 |
| 72h 500ng/ml *T.d.* | 99 | 5.7241 |  |  | 9 |
| **Fig. 3C** |  |  | ANOVA/post hoc Fisher LSD test: compare selected pairs | *P <0.05 |  |
| 0h Control untreated *P.g.* | 2.0124 | 0.2542 |  |  | 9 |
| 0h 100pg/ml *P.g.* | 2.0134 | 0.2342 |  |  | 9 |
| 0h 50ng/ml *P.g.* | 2.0574 | 0.1532 |  |  | 9 |
| 0h 200ng/ml *P.g.* | 2.0744 | 0.2231 |  |  | 9 |
| 0h 500ng/ml *P.g.* | 2.0433 | 0.5143 |  |  | 9 |
| 24h Control untreated*P.g.* | 2.1241 | 0.2595 |  |  | 9 |
| 24h 100pg/ml *P.g.* | 2.3125 | 0.4364 |  |  | 9 |
| 24h 50ng/ml *P.g.* | 2.1424 | 0.2324 |  |  | 9 |
| 24h 200ng/ml *P.g.* | 2.2526 | 0.3868 |  |  | 9 |
| 24h 500ng/ml *P.g.* | 2.5923 | 0.2172 |  |  | 9 |
| 48h Control untreated*P.g.* | 2.9532 | 0.2032 |  |  | 9 |
| 48h 100pg/ml *P.g.* | 3.1863 | 0.2254 |  |  | 9 |
| 48h 50ng/ml *P.g.* | 3.2655 | 0.3512 |  |  | 9 |
| 48h 200ng/ml *P.g.* | 3.4571 | 0.2632 |  |  | 9 |
| 48h 500ng/ml *P.g.* | 3.4812 | 0.3821 |  |  | 9 |
| 72h Control untreated*P.g.* | 4.1538 | 0.2545 |  |  | 9 |
| 72h 100pg/ml *P.g.* | 4.2351 | 0.2324 |  |  | 9 |
| 72h 50ng/ml *P.g.* | 4.3387 | 0.2681 |  |  | 9 |
| 72h 200ng/ml *P.g.* | 4.4221 | 0.3621 |  |  | 9 |
| 72h 500ng/ml *P.g.* | 4.2762 | 0.2782 |  |  | 9 |
| **Fig. 3D** |  |  | ANOVA/post hoc Fisher LSD test: compare selected pairs | *P <0.05 |  |
| 0h Control untreated*T.d.* | 2.0344 | 0.3154 |  |  | 9 |
| 0h 100pg/ml *T.d.* | 2.0766 | 0.2013 |  |  | 9 |
| 0h 50ng/ml *T.d.* | 2.0341 | 0.1016 |  |  | 9 |
| 0h 200ng/ml *T.d.* | 2.0366 | 0.2012 |  |  | 9 |
| 0h 500ng/ml *T.d.* | 2.0285 | 0.3011 |  |  | 9 |
| 24h Control untreated*T.d.* | 2.1424 | 0.4021 |  |  | 9 |
| 24h 100pg/ml *T.d.* | 2.0124 | 0.3013 |  |  | 9 |
| 24h 50ng/ml *T.d.* | 2.1341 | 0.2023 |  |  | 9 |
| 24h 200ng/ml *T.d.* | 2.1885 | 0.1013 |  |  | 9 |
| 24h 500ng/ml *T.d.* | 2.3825 | 0.3011 |  |  | 9 |
| 48h Control untreated*T.d.* | 3.0241 | 0.2012 |  |  | 9 |
| 48h 100pg/ml *T.d.* | 2.8251 | 0.3015 |  |  | 9 |
| 48h 50ng/ml *T.d.* | 3.2414 | 0.3028 |  |  | 9 |
| 48h 200ng/ml *T.d.* | 3.5674 | 0.2545 |  |  | 9 |
| 48h 500ng/ml *T.d.* | 3.2641 | 0.1844 |  |  | 9 |
| 72h Control untreated*T.d.* | 4.6352 | 0.3821 |  |  | 9 |
| 72h 100pg/ml *T.d.* | 4.5217 | 0.2632 |  |  | 9 |
| 72h 50ng/ml *T.d.* | 4.2524 | 0.2531 |  |  | 9 |
| 72h 200ng/ml *T.d.* | 4.1714 | 0.3651 |  |  | 9 |
| 72h 500ng/ml *T.d.* | 4.4714 | 0.4231 |  |  | 9 |
| **Fig. 4A** | **(%)** |  | ANOVA/post hoc Fisher LSD test: compare selected pairs | *P <0.05 |  |
| 0h Control untreated *P.g.* | 0 | 0 |  |  | 9 |
| 0h 100pg/ml *P.g.* | 0 | 0 |  |  | 9 |
| 0h 50ng/ml *P.g.* | 0 | 0 |  |  | 9 |
| 0h 200ng/ml *P.g.* | 0 | 0 |  |  | 9 |
| 0h 500ng/ml *P.g.* | 0 | 0 |  |  | 9 |
| 12h Control untreated*P.g.* | 19.52 | 4.5321 |  |  | 9 |
| 12h 100pg/ml *P.g.* | 22.12 | 2.6656 |  |  | 9 |
| 12h 50ng/ml *P.g.* | 21.95 | 3.0212 |  |  | 9 |
| 12h 200ng/ml *P.g.* | 42.52 | 2.9879 |  |  | 9 |
| 12h 500ng/ml *P.g.* | 47.63 | 3.6546 |  |  | 9 |
| 24h Control untreated*P.g.* | 26.35 | 2.9885 |  |  | 9 |
| 24h 100pg/ml *P.g.* | 27.54 | 3.3213 |  |  | 9 |
| 24h 50ng/ml *P.g.* | 28.65 | 4.6561 |  |  | 9 |
| 24h 200ng/ml *P.g.* | 49.52 | 3.5122 |  |  | 9 |
| 24h 500ng/ml *P.g.* | 55.65 | 4.5321 |  |  | 9 |
| **Fig. 4B** | **(%)** |  | ANOVA/post hoc Fisher LSD test: compare selected pairs | *P <0.05 |  |
| 0h Control untreated*T.d.* | 0 | 0 |  |  | 9 |
| 0h 100pg/ml *T.d.* | 0 | 0 |  |  | 9 |
| 0h 50ng/ml *T.d.* | 0 | 0 |  |  | 9 |
| 0h 200ng/ml *T.d.* | 0 | 0 |  |  | 9 |
| 0h 500ng/ml *T.d.* | 0 | 0 |  |  | 9 |
| 12h Control untreated*T.d.* | 20.21 | 5.3213 |  |  | 9 |
| 12h 100pg/ml *T.d.* | 23.51 | 4.3213 |  |  | 9 |
| 12h 50ng/ml *T.d.* | 24.25 | 5.5218 |  |  | 9 |
| 12h 200ng/ml *T.d.* | 45.67 | 3.6546 |  |  | 9 |
| 12h 500ng/ml *T.d.* | 48.95 | 2.9846 |  |  | 9 |
| 24h Control untreated*T.d.* | 25.45 | 1.9725 |  |  | 9 |
| 24h 100pg/ml *T.d.* | 28.55 | 5.3115 |  |  | 9 |
| 24h 50ng/ml *T.d.* | 30.55 | 5.2874 |  |  | 9 |
| 24h 200ng/ml *T.d.* | 50.57 | 3.6822 |  |  | 9 |
| 24h 500ng/ml *T.d.* | 56.92 | 5.2132 |  |  | 9 |
| **Fig. 5C** | **p-value** | **fdr** | ANOVA/post hoc Fisher LSD test: compare selected pairs | *P <0.05  **P < 0.01 ***P < 0.001 |  |
| White fat cell differentiation (GO:0045444) | 4.85E-08 | 1.38E-03 |  |  | 3 |
| Fat cell differentiation (GO:0045600) | 4.79E-08 | 1.28E-01 |  |  | 3 |
| Brown fat cell differentiation (GO:0050873) | 4.58E-08 | 1.16E-02 |  |  | 3 |
| Lipid localization (GO:0010876) | 4.48E-07 | 1.25E-03 |  |  | 3 |
| Neutrophil chemotaxis (GO:0030593) | 4.35E-02 | 1.14E-02 |  |  | 3 |
| Lipid transport (GO:0006869) | 4.11E-02 | 1.26E-02 |  |  | 3 |
| Locomotory behavior (GO:0007626) | 3.15E-03 | 1.11E-01 |  |  | 3 |
| Cartilage development (GO:0051216) | 2.52E-01 | 1.26E-01 |  |  | 3 |
| Hyaluronan biosynthetic process (GO:0030213) | 2.48E-02 | 1.17E-04 |  |  | 3 |
| Hyaluronan metabolic process (GO:0030212) | 2.45E-05 | 1.25E-04 |  |  | 3 |
| Endochondral bone morphogenesis (GO:0060350) | 2.33E-02 | 1.15E-03 |  |  | 3 |
| Regulation of osteoblast proliferation (GO:0033688) | 2.25E-03 | 1.27E-04 |  |  | 3 |
| Endochondral bone growth (GO:0003416) | 1.54E-03 | 1.26E-04 |  |  | 3 |
| Regulation of ossification (GO:0030278) | 1.17E-04 | 1.12E-04 |  |  | 3 |
| Bone development (GO:0060348) | 1.15E-04 | 1.15E-04 |  |  | 3 |
| Bone morphogenesis (GO:0060349) | 1.36E-04 | 1.26E-04 |  |  | 3 |
| Defense response (GO:0006952) | 0.98E-03 | 1.15E-04 |  |  | 3 |
| Cell chemotaxis (GO:0060326) | 0.85E-03 | 1.11E-04 |  |  | 3 |
| Immune effector process (GO:0002252) | 0.84E-02 | 1.22E-04 |  |  | 3 |
| Negative regulation of lipid storage (GO:0010888) | 0.87E-01 | 1.18E-04 |  |  | 3 |
| **Fig. 5D** |  |  | ANOVA/post hoc Fisher LSD test: compare selected pairs | *P <0.05  **P < 0.01 ***P < 0.001 |  |
| TLR2 RNAseq | 1.95E-02 |  |  |  | 3 |
| TLR2 qPCR | 1.62E-04 |  |  |  | 9 |
| TLR4 RNAseq | 1.87E-03 |  |  |  | 3 |
| TLR4 qPCR | 1.76E-02 |  |  |  | 9 |
| IL-6 RNAseq | 2.83E-04 |  |  |  | 3 |
| IL-6 qPCR | 2.64E-01 |  |  |  | 9 |
| IL-8 RNAseq | 3.25E-04 |  |  |  | 3 |
| IL-8 qPCR | 3.12E-03 |  |  |  | 9 |
| IL-1β RNAseq | 2.71E-04 |  |  |  | 3 |
| IL-1β qPCR | 2.58E-02 |  |  |  | 9 |
| **Fig. S1**  **CFU - S. mutans** |  |  |  |  |  |
| **Time (h)** | **Log10CFU/ml** | **STDEV** |  |  |  |
| 0 | 5.4 |  |  |  | 6 |
| 2 | 5.8 |  |  |  | 6 |
| 4 | 6.2 |  |  |  | 6 |
| 6 | 7.2 |  |  |  | 6 |
| 8 | 8 |  |  |  | 6 |
| 10 | 8.4 |  |  |  | 6 |
| 12 | 8.7 |  |  |  | 6 |
| 14 | 8.8 |  |  |  | 6 |
| 16 | 8.7 |  |  |  | 6 |
| 18 | 8.8 |  |  |  | 6 |
| 20 | 8.8 |  |  |  | 6 |
| **CFU - S. anginosus** |  |  |  |  |  |
| **Time (h)** | **Log10 CFU/ml** | **STDEV** |  |  |  |
| 0 | 5.132 | 0.121 |  |  | 6 |
| 2 | 5.425 | 0.132 |  |  | 6 |
| 4 | 5.825 | 0.352 |  |  | 6 |
| 6 | 6.888 | 0.245 |  |  | 6 |
| 8 | 7.863 | 0.223 |  |  | 6 |
| 10 | 8.124 | 0.115 |  |  | 6 |
| 12 | 8.525 | 0.228 |  |  | 6 |
| 14 | 8.782 | 0.124 |  |  | 6 |
| 16 | 8.933 | 0.123 |  |  | 6 |
| 18 | 9.251 | 0.325 |  |  | 6 |
| 20 | 9.552 | 0.211 |  |  | 6 |
| **CFU - P. intermedia** |  |  |  |  |  |
| **Time (h)** | **Log10 CFU/ml** | **STDEV** |  |  |  |
| 0 | 5.123 | 0.132 |  |  | 6 |
| 2 | 5.452 | 0.365 |  |  | 6 |
| 4 | 5.555 | 0.269 |  |  | 6 |
| 6 | 6.545 | 0.322 |  |  | 6 |
| 8 | 7.344 | 0.365 |  |  | 6 |
| 10 | 8.321 | 0.332 |  |  | 6 |
| 12 | 8.485 | 0.441 |  |  | 6 |
| 14 | 8.623 | 0.288 |  |  | 6 |
| 16 | 8.811 | 0.215 |  |  | 6 |
| 18 | 8.985 | 0.223 |  |  | 6 |
| 20 | 8.922 | 0.322 |  |  | 6 |
| **CFU – F. nucleatum** |  |  |  |  |  |
| **Time (h)** | **Log10 CFU/ml** | **STDEV** |  |  |  |
| 0 | 5.255 | 0.255 |  |  | 6 |
| 2 | 5.6321 | 0.375 |  |  | 6 |
| 4 | 5.921 | 0.453 |  |  | 6 |
| 6 | 6.554 | 0.251 |  |  | 6 |
| 8 | 7.526 | 0.333 |  |  | 6 |
| 10 | 7.622 | 0.354 |  |  | 6 |
| 12 | 8.556 | 0.297 |  |  | 6 |
| 14 | 8.628 | 0.288 |  |  | 6 |
| 16 | 8.821 | 0.386 |  |  | 6 |
| 18 | 8.844 | 0.455 |  |  | 6 |
| 20 | 8.975 | 0.312 |  |  | 6 |

| **CFU – P. gingivalis** |  |  |  |  |  |
| --- | --- | --- | --- | --- | --- |
| **Time (h)** | **Log10 CFU/ml** | **STDEV** |  |  |  |
| 0 | 5.235 | 0.122 |  |  | 6 |
| 2 | 5.822 | 0.254 |  |  | 6 |
| 4 | 6.122 | 0.422 |  |  | 6 |
| 6 | 6.875 | 0.213 |  |  | 6 |
| 8 | 7.521 | 0.151 |  |  | 6 |
| 10 | 8.222 | 0.302 |  |  | 6 |
| 12 | 8.663 | 0.210 |  |  | 6 |
| 14 | 8.725 | 0.225 |  |  | 6 |
| 16 | 9.142 | 0.113 |  |  | 6 |
| 18 | 9.113 | 0.355 |  |  | 6 |
| 20 | 9.128 | 0.221 |  |  | 6 |
| **CFU – T. denticola** |  |  |  |  |  |
| **Time (h)** | **Log10 CFU/ml** | **STDEV** |  |  |  |
| 0 | 5.357 | 0.223 |  |  | 6 |
| 2 | 5.455 | 0.205 |  |  | 6 |
| 4 | 5.921 | 0.125 |  |  | 6 |
| 6 | 6.527 | 0.322 |  |  | 6 |
| 8 | 6.632 | 0.245 |  |  | 6 |
| 10 | 7.505 | 0.175 |  |  | 6 |
| 12 | 7.854 | 0.286 |  |  | 6 |
| 14 | 8.557 | 0.225 |  |  | 6 |
| 16 | 8.854 | 0.321 |  |  | 6 |
| 18 | 8.831 | 0.332 |  |  | 6 |
| 20 | 8.921 | 0.432 |  |  | 6 |
